# Supplementary material for: Effectiveness and Safety of Ayurvedic Medicines in Type 2 Diabetes Mellitus Management: A Systematic Review and Meta-Analysis
Source: Front Pharmacol. 2022 Jun 8;13:821810. doi: 10.3389/fphar.2022.821810 (PMC9213670; doi:10.3389/fphar.2022.821810)
Supplement: Supplementary file 1 [file DataSheet1.zip › Supplementary Material/Forest and Funnel Plots/Gynostemma pentaphyllum/Insulin sensitivity.pdf]

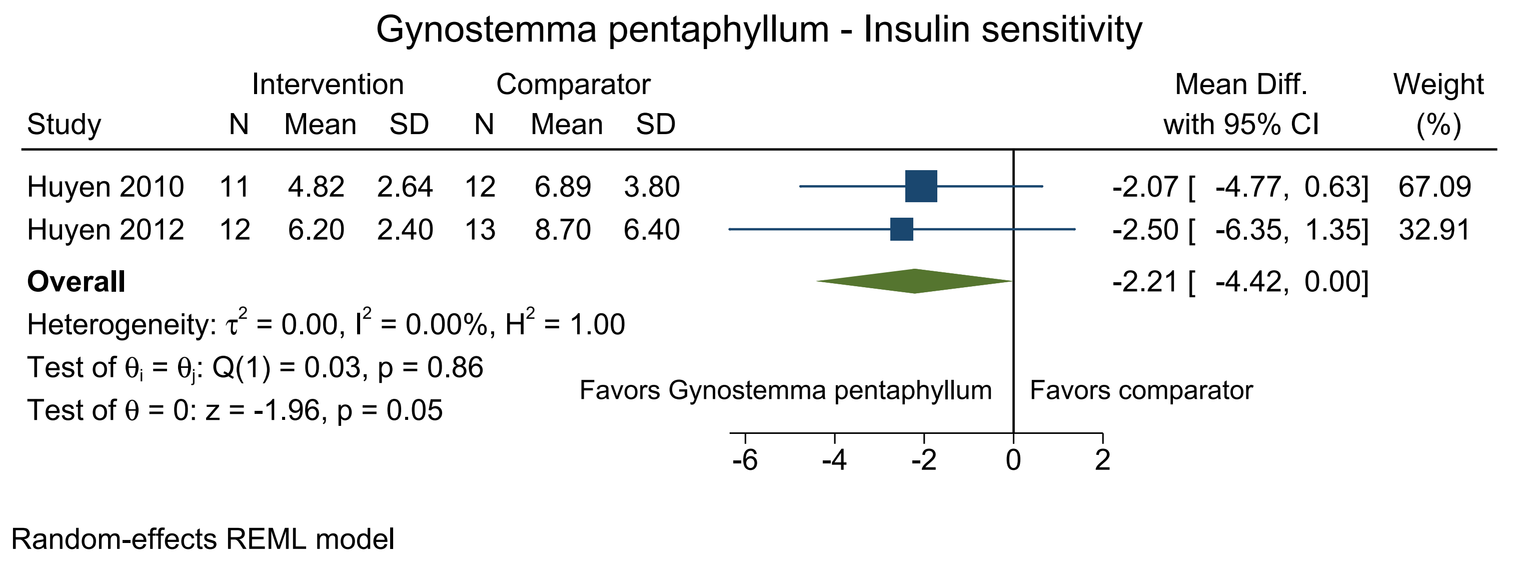
Gynostemma pentaphyllum - Insulin sensitivity
Intervention
Comparator
Study
N
Mean
SD
N
Mean
SD
Mean Diff. with 95% CI
Weight (%)
Huyen 2010 11 4.82 2.64 12 6.89 3.80 -2.07 [-4.77, 0.63] 67.09
Huyen 2012 12 6.20 2.40 13 8.70 6.40 -2.50 [-6.35, 1.35] 32.91
Overall -2.21 [-4.42, 0.00]
Heterogeneity: $\tau^2 = 0.00$, $I^2 = 0.00\%$, $H^2 = 1.00$
Test of $\theta_i = \theta_j$: Q(1) = 0.03, p = 0.86
Test of $\theta = 0$: z = -1.96, p = 0.05
Favors Gynostemma pentaphyllum
Favors comparator
-6
-4
-2
0
2
Random-effects REML model
